# Audiological findings in the Charcot-Marie-Tooth Disease

**DOI:** 10.1590/S1808-86942011000100023

**Published:** 2015-10-19

**Authors:** Erika Fukushima, Patricia Maria Sens, Ernani Lambert

**Affiliations:** 1 FCMSCSP. Assisting physician at the Otorhinolaryngology Unit of the Municipal Public Servant's Hospital (Hospital do Servidor Público Municipal); 2 Doctoral student, physician; 3 Otorhinolaryngologist, physician - private office

**Keywords:** charcot-marie-tooth disease, hearing loss, polyneuropathies

## INTRODUCTION

Charcot-Marie-Tooth disease (CMT) is one of a group of clinically and genetically heterogeneous polyneuropathies. The prevalence is 1:2,500. It is characterized by peripheral nerve degeneration, which results in distal muscle atrophy, loss of sensitivity, and hand and foot deformities; deep reflexes are also affected.^1,^^2^ The most common form is autosomal dominant.^3^

A clinically distinct condition is the association of this disease with hearing loss, suggesting an abnormal expression on the PMP22 gene.^1^

## OBJECTIVE

The purpose of this study was to report a case of a patient with Charcot-Marie-Tooth disease, highlighting its importance in the differential diagnosis of hearing loss.

## CASE REPORT

A female patient aged 42 years complained of difficulties in understanding speech, especially in noise environments, developing within the last 2 years. An electromyography was done in 1992 because of pain associated with absent deep reflexes in the lower limbs, and valgus knees; this test revealed marked chronic mixed peripheral axonal-demyelinating polyneuropathy (sensitive and motor) in the four limbs, which suggested type 1 CMT disease.

The pain progressed and affected the upper limbs and right shoulder; lower limb hypoesthesia, paresthesia and weakness were also present.

The otorhinolaryngological physical examination was normal. Auditory thresholds were within normal limits. Acoustic immittance testing demonstrated type A tympanograms, and bilaterally absent ipsilateral and contralateral acoustic reflexes. Responses were bilaterally absent in the brainstem auditory evoked potential (BAEP) test. Otoacoustic emissions were altered, and an assessment of auditory processing revealed decoding disorders and non-verbal deficits.

DNA studies of the patient and her parents (PCR and Southern blot methods) found no duplication or mutation on the PMP22 gene in the chromosome 17 (which explains most cases of type 1 CMT); these studies, however, do not preclude another form of CMT disease.

## DISCUSSION

Musiek et al.^2^ reported a similar case in which the only complaint was difficulty in understanding speech; the tests yielded similar results to our case, suggesting that an association between hearing loss and CMT disease is rare. However, we found reports in the literature of families diagnosed with CMT disease, including family members with hearing loss; the attending physicians were advised to carry out auditory testing for patients with CMT disease.^3,^^4^ In these families, the onset of hearing loss occurred at an earlier age in subsequent generations.

There is no single reported feature for hearing loss in CMT disease. Kovach et al.4 described sensorineural tests of limbs in a family, which ranged from normal results to profound losses. Papadakis et al.3 described a case of sudden hearing loss with profound bilateral sensorineural loss associated with CMT disease.

As with our patients, several authors reported altered acoustic reflexes and abnormal BAEP, in which wave latencies were increased or absent.^4^, ^5^, ^6^

Kovach et al.^1^ found altered BAEP and otoacoustic emissions results in the study family, which were similar to those in our case, suggesting that there are audiological differences between families with CMT disease and dysacusis. The mechanism of cochlear involvement, however, remains unclear. Starr et al.^6^ reported 8 subjects with CMT disease that presented absent BAEP and normal otoacoustic emissions, which were characterized as auditory neuropathy.^5^

As our case had altered otoacoustic emissions, the diagnosis of auditory neuropathy did not apply; however, BAEP and otoacoustic emissions changes were probably due to demyelinated auditory pathways because of CMT disease.

## COMMENTS

CMT disease is relevant in the differential diagnosis of sensorineural hearing loss, particularly if hereditary hearing loss is suspected. Specialists should be aware of the possible association between hearing loss and CMT disease, to support appropriate treatment for patients.
